# Mo_3_P/Mo heterojunction for efficient conversion of lithium polysulfides in high-performance lithium-sulfur batteries

**DOI:** 10.3389/fchem.2024.1459324

**Published:** 2024-08-12

**Authors:** Zhongpeng Sun, Yuanhao Wang, Jie Xu, Xia Wang

**Affiliations:** College of Physics, Qingdao University, University-Industry Joint Center for Ocean Observation and Broadband Communication, Qingdao, China

**Keywords:** lithium-sulfur batteries, three-dimensional ordered porous (3DOP) architecuture, heterogeneous structure, Mo_3_P, Mo

## Abstract

Realizing efficient immobilization of lithium polysulfides (LiPSs) as well as reversible catalytic conversion between LiPSs and the insoluble Li_2_S is vital to restrain the shuttle effect, which requires highly reactive catalysts for high-performance Li-S batteries. Here, three-dimensional ordered porous Mo-based metal phosphides (3DOP Mo_3_P/Mo) with heterogeneous structures were fabricated and utilized as separator-modified coatings for Li-S batteries to catalyze the conversion of LiPSs. The adsorption, catalytic and electrochemical performance of the corresponding cells were compared among 3DOP Mo_3_P/Mo and 3DOP Mo, by kinetic and electrochemical performance measurements. It was found that the cell with 3DOP Mo_3_P/Mo modified separator deliver better electrochemical performance, with a high specific capacity of 469.66 mAh g^−1^ after 500 cycles at a high current density of 1°C. This work provides an idea and a guideline for the design of the separator modification for high-performance Li-S batteries.

## Introduction

With the shortage of non-renewable energy sources such as traditional fossil fuels, the development of electrochemical energy storage systems with high energy density and long life is the key to increase the utilization of renewable energy sources. In recent years, lithium-sulfur (Li-S) batteries have received extensive research attention due to their high theoretical energy density (2,600 Wh kg^−1^), cost-effectiveness, and environmental friendliness of sulfur (Robinson et al., 2021). However, Li-S batteries still face many obstacles, including the slow kinetics of lithium polysulfides (LiPSs) conversion during charging and discharging to cause the shuttling effect (Wang et al., 2023), associated with the volume expansion caused by sulfur phase transition. It is pivotal to address the above issues for accelerating the development of Li-S batteries (Wang et al., 2022; Chen et al., 2024).

Electrocatalysts have been reported to promote the conversion of LiPSs to improve the utilization of sulfur and cycling stability (Dai et al., 2021; Feng et al., 2022). Among the numerous catalysts, transition metal phosphides (TMPs) (Liu et al., 2023; Zhang et al., 2024), such as CoP (Sun et al., 2023), Ni_2_P (Niu et al., 2023), etc. have been widely utilized in Li-S batteries to catalyze the reaction of LiPSs due to their good structural and thermal stability, along with abundant active sites. Especially, Mo_3_P possesses low work function accompanied by rich valence electrons in 3d orbitals, thus presenting very broad application prospects in boosting the conversion of LiPSs, which has scarcely been reported.

Generally, the conductivity of the molybdenum phosphide as a semiconductor material can not meet the requirements of an electrocatalyst perfectly (Feng et al., 2024). The combination of the molybdenum phosphide and materials good electrical conductivity, such as carbon, metal, etc. to form composited or heterogeneous materials has been regarded as an effective strategy to improve the conductivity and promote charge transfer (Yuan et al., 2017; Feng et al., 2023). For example, Li et al. designed the Co-CoP heterostructure to effectively confine and catalyze the conversion of LiPSs while enhancing ionic and electron migration. The volume expansion of LiPSs on NCNT@Co-CoP-1 was also significantly reduced at high S loading, and the catalytic conversion efficiency was improved (Li et al., 2021). Therefore, the design of metal phosphide/metal heterojunctions is crucial to further improve the performance of Li-S batteries.

In this work, we report for the first time the synthesis of three-dimensional ordered porous (3DOP) Mo_3_P/Mo composites as separator-modified coatings by a sol-gel method to improve the electrochemical performance of Li-S batteries (Li et al., 2021; Li et al., 2021; Zhu et al., 2021). The material characterization, adsorption performance, and battery performance tests show that compared with the monometallic (Mo) modified separator, the heterostructured Mo_3_P/Mo catalyst possesses a stronger interaction with Li_2_S_6_, which effectively restricts the shuttling effect of LiPSs, strengthens the electron transfer ability, improves the reaction kinetics of LiPSs, and further enhances the sulfur utilization, thus improving the cycling stability of Li-S batteries.

## Experimental section

### Preparation of 3DOP Mo_3_P/Mo

Firstly, 0.741 g of (NH_4_)_2_MoO_4_ were dissolved in 5 mL of deionised water (DI) under magnetic stirring, and then 0.0792 g of diammonium hydrogen phosphate [(NH_4_)_2_HPO_4_] was added. Subsequently, 0.252 g of C_6_H_5_NO_4_ was added in the above solution and placed in an oil bath under 90°C until the solution became a gel. Afterwards, 1.56 g of SiO_2_ was mixed with the gel, which was then dried in an oven. The dried sample was then calcined at 900°C for 6 h under Ar/H_2_ gas. The final 3DOP structured product was obtained through etching SiO_2_ using NaOH solution.

### Preparation of 3DOP Mo

Firstly, 0.7062 g of (NH_4_)_2_MoO_4_ were dissolved in 15 mL of deionised water (DI) under magnetic stirring, and then 2.446 g of C_6_H_5_NO_4_ was added in the above solution and placed in an oil bath under 90°C until the solution became a gel. Afterwards, 1.56 g of SiO_2_ was mixed with the gel, which was then dried in an oven. The dried sample was then calcined at 900°C for 8 h under Ar/H_2_ gas. The final 3DOP structured product was obtained through etching SiO_2_ using NaOH solution.

### Materials characterization

The phase and purity of the resulting samples were analyzed by powder x-ray diffraction (XRD) (Rigaku D/Max X-ray diffractometer with Cu Kα radiation). Morphological images of the final products were obtained by scanning electron microscopy (SEM, JSM-6700F, JEOL). The surface elemental composition and chemical valence states of the product were determined by X-ray photoelectron spectroscopy (XPS, PHI-5702, Physical Electronics).

### Synthesis of sulfur electrodes

The mixture of Ketjen black and sulfur powder with a mass ratio of 7:3 was ground for 30 min and heated at 155°C for 12 h under an Ar atmosphere. After cooling to room temperature, the sulfur cathode with 65% sulfur was obtained. Subsequently, the working electrodes named KB/S were prepared through casting the above homogeneous slurry, composed of KB/S, KB, and LA133 with a mass ratio of 7:2:1, on Al foil, followed by vacuum dried overnight at 60°C. Finally, the Al foil coated with KB/S was punched into disks with a diameter of 12 mm.

### Assembly of Li-S batteries

Li-S cells were assembled using CR2032-type coin cells in the Ar-filled glove box employing the KB/S electrode as the cathode, Li as the anode, Celgard 2,400 as the separator, 3DOP Mo_3_P/Mo as the separator interlayer and 1.0 M LiTFSI in a DME/DOL (v/v = 1/1) solution with 1 wt% LiNO_3_ as the electrolyte. The areal sulfur loading of the cathode was around 1.3 mg cm^−2^ (E/S ratio: 50 μL mg^−1^).

### Electrochemical characterizations

Galvanostatic charge/discharge tests for Li-S cells wereacquired with the voltage ranging from 1.8 to 2.7 V (Neware, Shenzhen, China). Cyclic voltammetry (CV) and electrochemical impedance (EIS) measurements were collected on a CHI660 electrochemical workstation. And the CV profiles were conducted at a scanning rate of 0.1 mV s^−1^ from 1.8 to 2.7 V. Electrochemical impedance spectroscopy (EIS) data were performed by the same electrochemical workstation by applying a 1 mV amplitude signal from 100 kHz to 0.01 Hz. The galvanostatic intermittent titration technique (GITT) were conducted at a pulse current of 0.05°C, and the pulse time and relaxation time are both 30 min.

### Adsorption test of LiPSs

Firstly, Li_2_S_6_ solution (2 mM) was obtained through vigorously stirring the mixed solution consisting of Li_2_S and S dissolved in DOL + DME (v/v, 1:1) solvent with a molar ratio of 1:5. Afterwards, 3DOP Mo_3_P/Mo and 3DOP Mo were separately placed into the above Li_2_S_6_ solution with 1 mL. After 6 h of adsorption, the supernatants were tested by ultraviolet spectrophotometer, and the four catalysts were measured using XPS.

## Results and discussion

**FIGURE 1:**
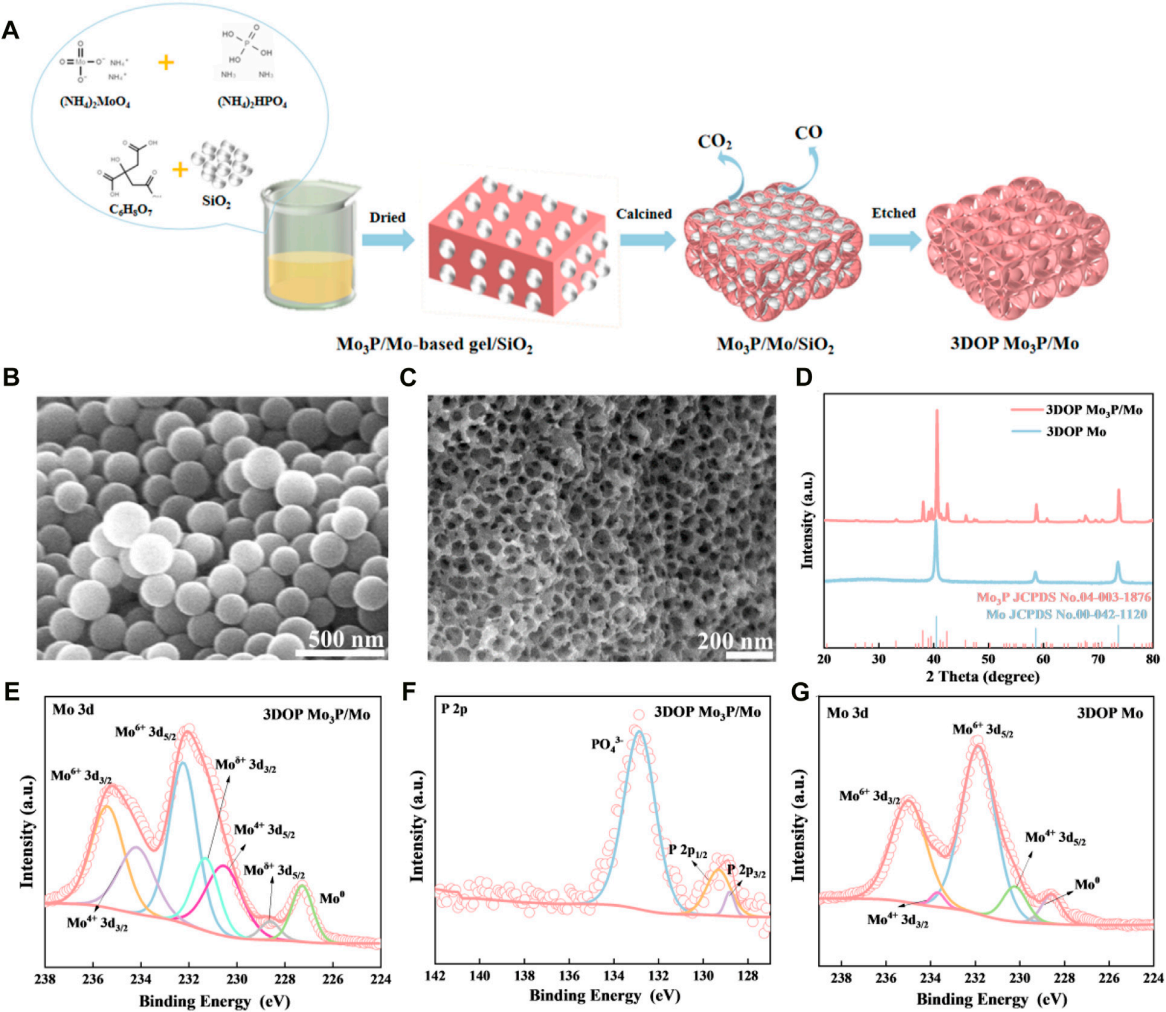
**(A)** Scheme of the synthesis process of 3DOP Mo_3_P/Mo. **(B, C)** SEM images of SiO_2_ templates and 3DOP Mo_3_P/Mo, respectively. **(D)** XRD patterns of 3DOP Mo_3_P/Mo and 3DOP Mo. High resolution XPS spectra of **(E)** Mo 3d and **(F)** P 2p in 3DOP Mo_3_P/Mo. **(G)** High resolution XPS spectra of Mo 3d.

The novel 3DOP Mo_3_P/Mo was fabricated and corresponding synthesized process was depicted in Figure 1A. Firstly, ammonium molybdate was mixed with diammonium hydrogen phosphate and then citric acid and SiO_2_ spheres with average diameter size of around 150 nm (Figure 1B) were added to form a gel under oil bath, which was subsequently calcined to generate a Mo_3_P/Mo/SiO_2_ composite. After etching of SiO_2_, the final sample was achieved with 3D ordered hierarchical porous architecture (Wang et al., 2018; Li et al., 2021), composed of ordered macropores with a diameter size of approximately 150 nm, consistent with SiO_2_ spheres, and numerous meso-micropores caused by the decomposition of citric acid to generate gases, which was confirmed by the SEM image (Figure 1C).

Subsequently, the powder XRD patterns of the prepared 3DOP samples are shown in Figure 1D. All diffraction peaks of the prepared product are ascribed to Mo_3_P (JCPDS No. 04-003-1876) and Mo (JCPDS No. 00-042-1120) in the absence of other impurities, revealing the formation of pure Mo_3_P/Mo phase. Besides, the corresponding monometallic as comparison sample is also pure Mo (JCPDS No. 00-042-1120).

Additionally, the survey XPS spectrum (Supplementary Figure S1A) shows that the surface elements are mainly composed of Mo, P, C and O in the 3DOP Mo_3_P/Mo material (Wu et al., 2023). The Mo 3d high-resolution scans, as shown in Figure 1E, the four main peaks presented at 235.4, 234.1, 232.2, and 230.5 eV belong to Mo^6+^ and Mo^4+^, which are oxidized phases due to the oxidation of the 3DOP Mo_3_P/Mo surface. The pair of peaks with binding energies of 231.3 and 228.6 eV corresponds to Mo^δ+^ (0<δ < 4) of Mo_3_P and the peak at 227.3 eV is attributed to Mo^0^. Meanwhile, the high resolution XPS spectrum of P 2p (Figure 1F) displays three main peaks at 132.9, 129, and 128 eV, belonging to PO_4_^3-^, 2p_1 /2_ and 2p_3 /2._ In addition, the full spectrum and the valence distribution of 3DOP Mo are shown in Supplementary Figures S1B, G. The surface elements of the 3DOP Mo materials are mainly composed of Mo, C and O. There are four main peaks at 234.9, 233.6, 231.8, and 230.3 belonging to Mo^6+^ and Mo^4+^, respectively, which are due to the surface oxidation in the high resolution Mo 3d spectrum, while the characteristic peak at around 228.2 eV corresponds to Mo^0^.

**FIGURE 2:**
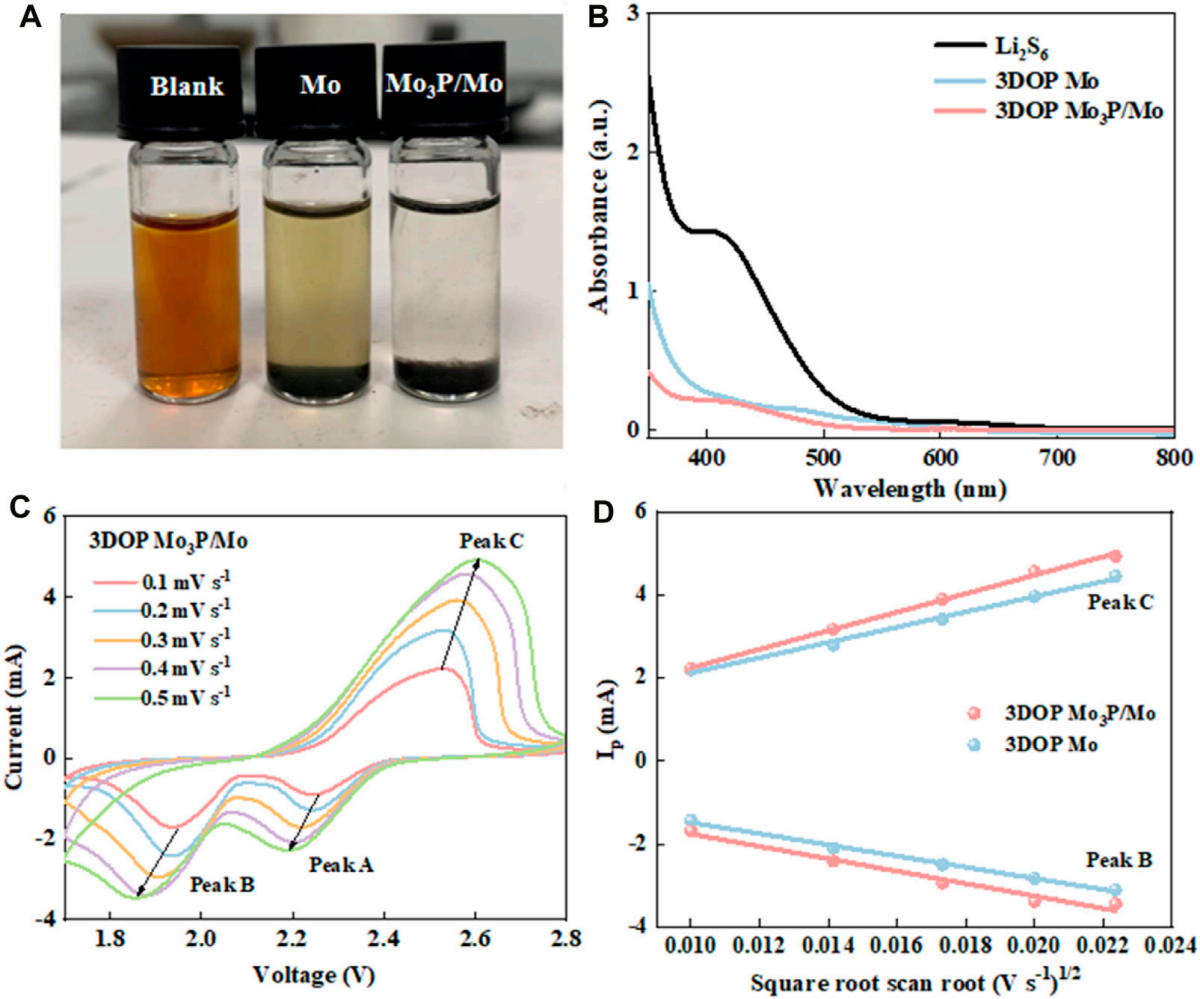
**(A, B)** Static lithium polysulfide adsorption tests and UV-vis absorption spectra of Li_2_S_6_ solutions after adding 3DOP Mo_3_P/Mo and 3DOP Mo. **(C)** CV curves of 3DOP Mo_3_P/Mo under different sweep speeds. **(D)** Linear fitting of peak currents *versus* square root scan rate of the as-synthesized catalysts.

In order to resolve the shuttle effect of LiPSs, the catalysts firstly are expected to be capable of achieving strong adsorption. Accordingly, in view of investigating the adsorption capability of 3DOP Mo_3_P/Mo and 3DOP Mo, the adsorption visualization measurements for two samples and Li_2_S_6_ solutions (Wang et al., 2022; Huang et al., 2023). As shown in Figure 2A, the initial yellow color of Li_2_S_6_ solution becomes light for 3DOP Mo material, while the yellow Li_2_S_6_ solution contained 3DOP Mo_3_P/Mo changes to nearly transparent after 6 h, suggesting the stronger adsorption capability of 3DOP Mo_3_P/Mo towards LiPSs (Lei et al., 2018). Moreover, the solutions after visualization adsorption experiments were diluted and then measured using a UV–vis spectroscopy. The UV–vis spectra in Figure 2B show a lower peak of 3DOP Mo_3_P/Mo compared with 3DOP Mo at around 420 nm, demonstrating the above conclusion (Xu et al., 2024).

The reaction kinetics of these two samples about the conversion of LiPSs were also further explored. Figure 2C and Supplementary Figure S2 show the CV curves of Li-S batteries assembled by 3DOP Mo_3_P/Mo modified separator at different scan rates. In addition, the diffusion coefficients of each electrode were calculated based on these CV curves in Supplementary Figure S3 (Wang et al., 2022), which were calculated with the classical Randles-Sevcik Equation:

$$I_{p}=2.69\times 10^{5}n^{3/2}AD_{{Li}^{+}}^{\;1/2}C_{{Li}^{+}}v^{1/2}$$ (1)

Thereinto, *I*_*p*_ and *n* represent the peak current and the number of electrons transferred, respectively. *A* is the active area of the electrode, *D*_*Li+*_ and *C*_*Li+*_ express the diffusion coefficient and concentration of Li^+^, respectively, while v is the scan rate. According to the above formulation, the Li^+^ diffusion coefficient is a positive correlation with the slopes of *v* ^*1/2*^ and *I*_*p*_ (Xu et al., 2022). As shown in Figure 2D, the Li-S battery assembled by 3DOP Mo_3_P/Mo possesses the highest slope in both oxidation and reduction reactions, which suggests that 3DOP Mo_3_P/Mo promotes charge transfer and significantly accelerates redox reaction kinetics (Wu et al., 2023).

**FIGURE 3:**
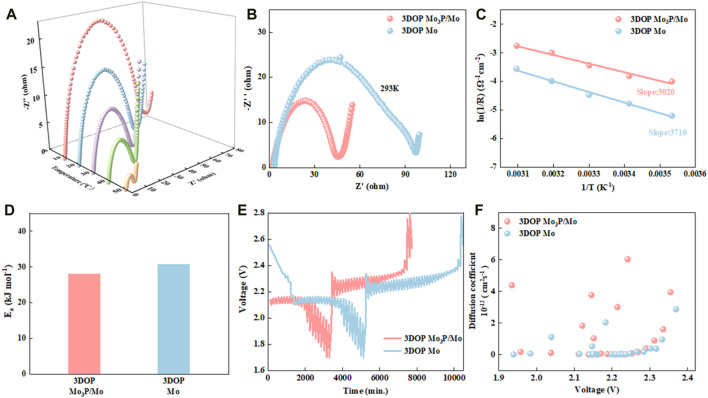
**(A)** EIS spectra of 3DOP Mo_3_P/Mo at different temperatures. **(B)** Electrochemical impedance spectroscopy at 293 K. **(C)** Arrhenius plots of ln (1/*R*_*t*_) vs.1/*T* in 3DOP Mo_3_P/Mo and 3DOP Mo-based batteries. **(D)** Activation energy (*Ea*) of 3DOP Mo_3_P/Mo and 3DOP Mo. **(E)** GITT curves of 3DOP Mo_3_P/Mo and 3DOP Mo at 40 mA g^−1^. **(F)** Li^+^ diffusion coefficients of 3DOP Mo_3_P/Mo and 3DOP Mo.

To clarify the reason for good reaction dynamics of 3DOP Mo_3_P/Mo, the electrochemical impedance spectroscopy (EIS) spectra of 3DOP Mo_3_P/Mo and 3DOP Mo under various temperatures were measured, depicted in Figure 3A and Supplementary Figure S4 (Jiang et al., 2022). As is observed that all Nyquist plots mainly consist of a semicircle in the mid/high-frequency region as well as a straight line in the low-frequency region, corresponding to the charge transfer resistance (*R*_*ct*_) as well as the ion diffusion impedance, respectively. In contrast with 3DOP Mo, 3DOP Mo_3_P/Mo delivers a much smaller semicircle at each temperature (Figure 3B) (Hao et al., 2020; Zhao et al., 2020). Besides, activation energy (*Ea*) of 3DOP Mo_3_P/Mo and 3DOP Mo materials required for chemical reactions was analyzed on the basis of Arrhenius equations (Figures 3C, D). 3DOP Mo_3_P/Mo exhibits a lower *E*_*a*_ value of approximately 28.04 kJ mol^−1^, in comparison with 3DOP Mo (30.84 kJ mol^−1^), revealing that 3DOP Mo_3_P/Mo is beneficial to speeding up the conversion reaction. In addition, the diffusion kinetics of Na^+^ in 3DOP Mo_3_P/Mo and 3DOP Mo was also further evaluated through galvanostatic intermittent titration technique (GITT) (Wang et al., 2023). Figures 3E, F depict the GITT time-potential curves at 40 mA g^−1^ with the corresponding Na^+^ diffusion coefficients. As is clearly observed that the Na^+^ diffusion coefficients (*D*_Li+_) of 3DOP Mo_3_P/Mo (6.033 × 10^−12^ - 3.387 × 10^−16^ cm^2^ s^−1^) are larger than those of 3DOP Mo (2.86 × 10^−12^ - 2.61 × 10^−16^ cm^2^ s^−1^), indicating that Li^+^ is more beneficial to diffusing in 3DOP Mo_3_P/Mo (Qi et al., 2023).

**FIGURE 4:**
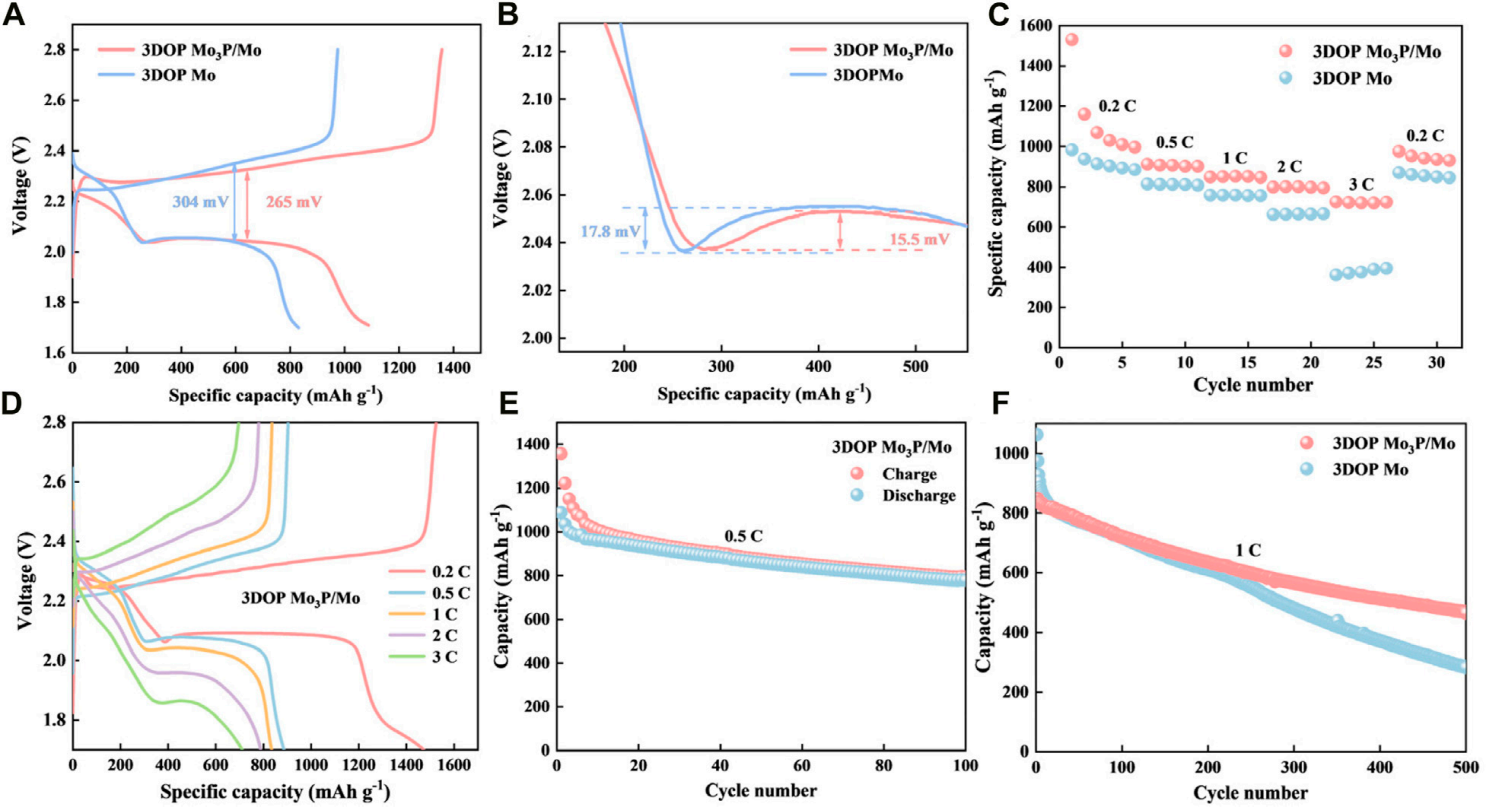
**(A, B)** Discharge–charge curves. **(C, D)** Rate performance of 3DOP Mo_3_P/Mo and 3DOP Mo for Li-S batteries. **(E)** Cycling performances at 0.5°C of 3DOP Mo_3_P/Mo. **(F)** Cycling performances at 1°C of 3DOP Mo_3_P/Mo and 3DOP Mo.

In order to investigate the electrochemical performance of Li-S batteries with 3DOP Mo_3_P/Mo and 3DOP Mo as materials for diaphragms applied to Li-S batteries, button batteries were assembled with different samples of modified separators and electrochemically relevant tests were performed. In order to further investigate the polarization phenomenon during the redox process and the potential barriers for nucleation and dissolution of Li_2_S, constant-current charge-discharge tests were carried out at a current density of 0.5 C. The charge-discharge curves are shown in Figure 4A (Zhou et al., 2020; Sun et al., 2024; Wu et al., 2024). The charge-discharge curves of the first cycle show that the two characteristic discharge platforms of the 3DOP Mo_3_P/Mo battery are higher and flatter than those of the 3DOP Mo, and the corresponding capacity is also larger. The polarization voltages of 3DOP Mo_3_P/Mo and 3DOP Mo cells were 265 mV and 304 mV (Tang et al., 2021; Zhao et al., 2021), respectively, which indicates that the 3DOP Mo_3_P/Mo catalyst could better promote the conversion of LiPSs, and this result was in agreement with the CV curve data. What’s more, the catalytic effect of 3DOP Mo_3_P/Mo can be further explained by scrutinizing the voltage magnification plots. In general, the potential difference between the initial voltage and the tangent of the potential plateau for the conversion of Li_2_S_4_ to Li_2_S_2_ correlates with the ease of generating insoluble Li_2_S_2_/Li_2_S, meaning a smaller potential difference represents a more likely occurrence of the reaction (Wu et al., 2022; Song et al., 2022). Figure 4B shows a partial enlargement of the discharge curve, which represents the conversion of LiPSs to insoluble Li_2_S_2_/Li_2_S, where the voltage differences of 15.5 mV and 17.8 mV for 3DOP Mo_3_P/Mo and 3DOP Mo cells, respectively, which demonstrates that soluble LiPSs (Li_2_S_4_) is more susceptible to the reaction catalyzed by 3DOP Mo_3_P/Mo catalyzed is more easily converted to insoluble lithium sulfide (Li_2_S_2_/Li_2_S) (Sun et al., 2023; Chen et al., 2024; Xiang et al., 2024).

Figure 4C shows the rate performance of the cells corresponding to 3DOP Mo_3_P/Mo and 3DOP Mo. 3DOP Mo_3_P/Mo cells possess specific capacities of 1529.77, 912.2, 848.34, 797.79 和 724.95 mAh g^−1^ at current densities of 0.2°C, 0.5°C, 1°C, 2°C, and 3°C (Xu et al., 2022), respectively, and their specific capacities can still recover to 975.44 mAh g^−1^ when the current density returns to 0.2°C (Wang et al., 2023). When the current density was restored to 0.2°C, the specific capacity could still be restored to 975.44 mAh g^−1^, indicating that the 3DOP Mo_3_P/Mo cells deliver good rate performance compared to 3DOP Mo, which was attributed to the fact that 3DOP Mo_3_P/Mo could better promote the cell’s internal electrochemical processes. Figure 4D and Supplementary Figure S5 shows the charge/discharge curves of the two materials at different current densities (Guo et al., 2022), and even at high current densities, the 3DOP Mo_3_P/Mo cell still shows a basic reaction plateau, which again indicates a faster electrochemical reaction. Figure 4E shows that 3DOP Mo_3_P/Mo possesses high initial discharge and charge capacities of around 1357 and 1087 mAh g^−1^, respectively, and stable capacity of 795 mAh g^−1^ after 100 cycles at 0.5°C (Guan et al., 2023). In addition, even at a high current density of 1°C, 3DOP Mo_3_P/Mo provided a higher specific capacity of 469.66 mAh g^−1^ after 500 cycles compared to 3DOP Mo (283.33 mAh g^−1^) (Figure 4F) (Wu et al., 2023), which suggests that the metallic phosphides/Mo heterostructuredemonstrate long cycle life and high specific capacity.

## Conclusion

In summary, in this work, a three-dimensional porous Mo_3_P/Mo heterostructure was successfully designed as a novel multifunctional catalyst for Li-S batteries. 3DOP Mo_3_P/Mo possesses a strong adsorption for LiPSs and rapid transfer of internal electrons, which promoted the conversion of LiPS to effectively inhibit the shuttle effects. The results showed that the lithium-sulfur battery using 3DOP Mo_3_P/Mo as the diaphragm could provide a high reversible capacity of up to 469.6 mAh g^−1^ after 500 cycles at 1°C. This work presents a new idea for the design of multifunctional electrocatalysts with molybdenum-based heterostructures for lithium-sulfur battery.

## Data Availability

The datasets presented in this study can be found in online repositories. The names of the repository/repositories and accession number(s) can be found in the article/Supplementary Material.
