# Supplementary material for: Mo3P/Mo heterojunction for efficient conversion of lithium polysulfides in high-performance lithium-sulfur batteries
Source: Front Chem. 2024 Aug 12;12:1459324. doi: 10.3389/fchem.2024.1459324 (PMC11345131; doi:10.3389/fchem.2024.1459324)
Supplement: Supplementary file 1 [file DataSheet1.PDF]

Supporting Information

Mo_3P/Mo heterojunction for efficient conversion of lithium polysulfides in high-performance lithium-sulfur batteries

Zhongpeng Sun[1], Yuanhao Wang[1], Jie Xu[1], Xia Wang[1*]

[1]University-Industry Joint Center for Ocean Observation and Broadband Communication, College of Physics, Qingdao University, Qingdao, 266071, Shandong, China

[*]**Correspondence：**
Xia Wang[1] *
E-mail: wangxiakuaile@qdu.edu.cn

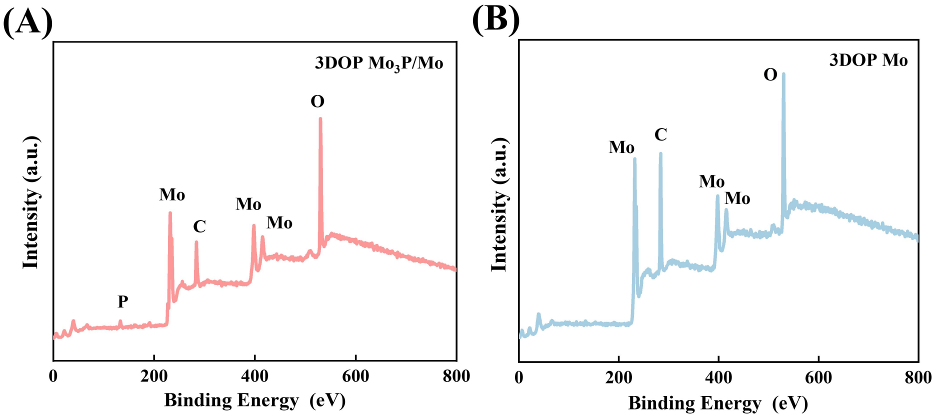

Supplementary Figure 1. XPS full spectrum of (A) 3DOP Mo_3P/Mo and (B) 3DOP Mo.

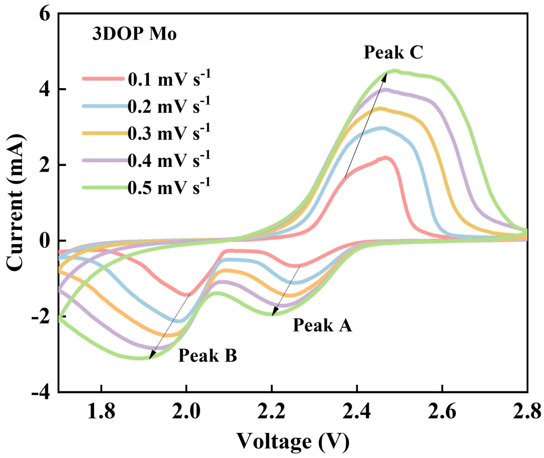

Supplementary Figure 2. CV curves at different sweep speeds of 3DOP Mo.

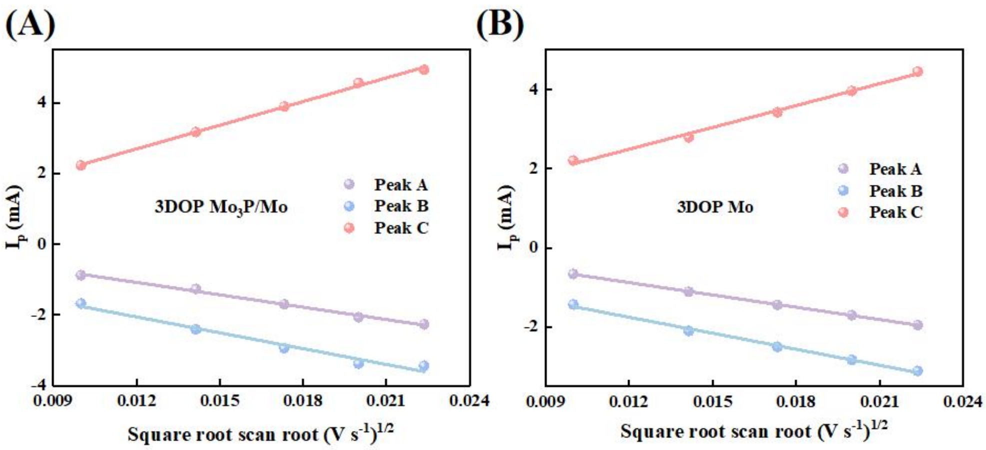

Supplementary Figure 3. Relationship between the peak current (I_p) and the square root of scan rate ($v^{1/2}$) of (A) 3DOP Mo_3P/Mo and (B) 3DOP Mo.

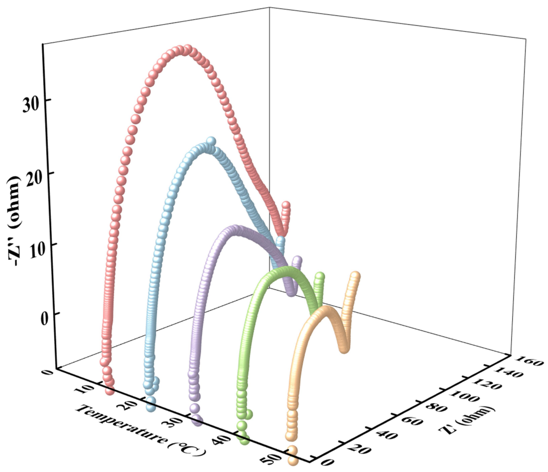

Supplementary Figure 4. EIS curves of 3DOP Mo at different temperatures.

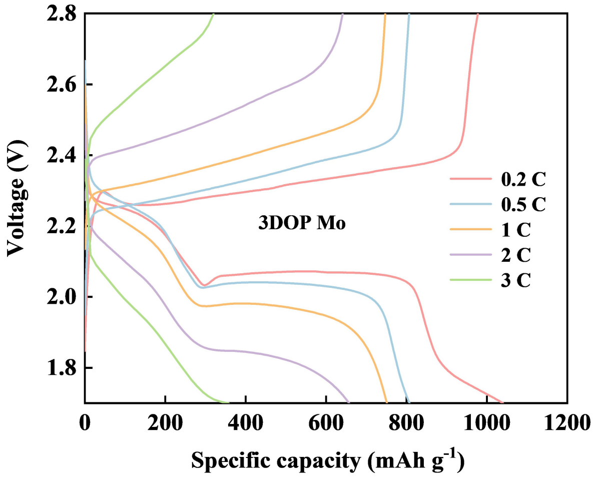

Supplementary Figure 5. Charge-discharge curves of 3DOP Mo at different current densities.
